# Supplementary material for: Individual-Level and Neighborhood-Level Factors Associated with Longitudinal Changes in Cardiometabolic Measures in Participants of a Clinic-Based Care Coordination Program: A Secondary Data Analysis
Source: J Clin Med. 2022 May 20;11(10):2897. doi: 10.3390/jcm11102897 (PMC9145991; doi:10.3390/jcm11102897)
Supplement: Supplementary file 1 [file jcm-11-02897-s001.zip › jcm-1730108-supplementary.pdf]

SUPPLEMENTARY MATERIALS

Supplementary File S1: Detailed methods for determining neighborhood-level social risks and driving time to PCP clinic location.

Supplementary Table S1 Results from Parsimonious model of Diastolic Blood Pressure outcome

Supplementary Table S2: Multivariable models of LDL cholesterol outcome for 2377 participants with pre-and post-intervention measures

Supplementary Table S3: Multivariable models of hemoglobin A1C outcome for 1290 participants with pre-and post-intervention measures

Supplementary Table S4: Multivariable models of systolic blood pressure outcome for 4618 participants with pre-and post-intervention measures

Supplementary Table S5: Multivariable models of diastolic blood pressure outcome for 4619 participants with pre-and post-intervention measures.

Supplementary Table S6: Solution for Random Effects by clinics- LDL outcome

Supplementary Table S7: Solution for Random Effects by clinics: HbA1C outcome

Supplementary Table S8: Solution for Random Effects by clinics: Systolic BP outcome

Supplementary Table S9: Solution for Random Effects by clinics: Diastolic BP outcome

Supplementary Figure S1: Interaction of female sex with pre-intervention systolic BP on adjusted change in systolic BP

Supplementary Figure S2: Interaction of female sex with pre-intervention systolic BP on adjusted change in diastolic BP

Supplementary Figure S3: Interaction of female sex with pre-intervention HbA1C on adjusted change in HbA1C

Supplementary File S1   Detailed methods for determining neighborhood-level social risks and driving time to PCP clinic location

Data used in the analysis were obtained from available secondary data and spatial analyses of de-identified patient address in combination with address-level datasets.

Geolocating patient records

To ascertain spatial locations and neighborhood (e.g., census tract) values for analyses, an extract of 9317 LIGHT^2 patient addresses was created containing only a unique patient ID and the patient address (Address, City, County, ZIP Code, and State). All personal health information variables were removed. Records were geocoded or translated from address information to geographic coordinates, providing latitude and longitude attributes. The address file was geocoded using ArcGIS Online World Geocoding Service, version dated June 6, 2019.[1]

Supplementary File1-Table 1 displays the geocoding precision of the resulting dataset. 6507 (69.84%) were located at the housing unit centroid level (PointAddress, Subaddress), 2691 were located at the street address level (StreetAddress), and 44 were located at the ZIP Code + 4 centroid level (PostalExt). The remaining 0.8% were located at the ZIP code centroid or street name levels. Of 9317

records, over 99% were located with the highest levels of accuracy. Definitions for the geocode precision types can be found by accessing the ESRI Geocoding Service Output documentation.[1]

Supplementary File S1-Table 1. Geocoding Precision		
**Geocode Precision**	**Count**	**Percentage**
PointAddress	6506	69.83
StreetAddress	2691	28.88
PostalExt	44	0.47
StreetName	43	0.46
StreetAddressExt	20	0.21
Postal	12	0.13
Subaddress	1	0.01

Census tract and census block group IDs were added to the resulting dataset by performing a spatial intersection between the geocoded results and the 2014 TIGER/Line Block Group Shapefile.[2] The resulting information was stored in a password-protected Microsoft SQL database for further analysis.

Poverty and education secondary- data elements

Analysis of variation in patient socio-economic status (SES) required a data source with sub-county variation; this was necessary as 60% of patients were identified as residing in a single county (Boone, Missouri). Data were obtained from the US Census Bureau 2010-2014 American Community Survey (ACS) 5-Year Estimates, accessed through the Census Bureau's data system.[3] The ACS is an annual, national survey that contains "social, economic, housing, and demographic characteristics" about the US population. Data are released as 5-year period estimates at units of geography down to the census block group level. From this dataset, 2 variables were selected for inclusion in the analysis: the percentage of census tract residents living below 200% of the federal poverty level (FPL) and the percentage of block group resident adults over age 25 without a high school diploma or equivalency. Percentages were calculated from tabulated data by the University of Missouri Center for Applied Research and Engagement Systems (CARES). Supplementary File1-Table 2 shows the variables used in the poverty calculation:

Supplementary **File S1-Table 2. Variables in Poverty Calculation**		
**Column ID**	**Table ID & Line Number**	**Definition**
0	B17024001	Universe:<br>Population (for whom poverty status is determined)
1	B17024003 - B17024010 +<br>B17024016 - B17024023 +<br>B17024029 - B17024036 +<br>B17024042 - B17024049 +<br>B17024055 - B17024062 +<br>B17024068 - B17024075 +<br>B17024081 - B17024088 +<br>B17024094 - B17024101 +<br>B17024107 - B17024114 +<br>B17024120 - B17024127	Numerator:<br>All Ages, Ratio of Income to Poverty Level: Under 2.00

Supplementary File1-Table 3 shows the variables used in the educational attainment calculation.

Supplementary **File S1-Table 3. Variables in Educational Attainment Calculation**		
**Column ID**	**Table ID & Line Number**	**Definition**
0	B15002001	Universe: Population 25 Years and Over
1	B15002003-B15002010 + B15002020-B15002027	Numerator: Age 25 and older; No schooling completed through 12th grade, no diploma

The resulting census-tract level poverty and census block group-level educational attainment estimates were added to the de-identified patient dataset by joining the 2 datasets on census tract and block-group IDs.

Distance data elements

Variables distance to the nearest grocery store/supermarket and distance to primary care physician clinic location are evaluated at the patient-address level. Distance variables were calculated using an ESRI origin-destination (OD) cost matrix network analysis (ESRI, 2018) using the StreetMap North America (ESRI, 2013) street network dataset.[4] Patient addresses were assigned as origins, with a search tolerance set to 1200 meters to ensure all records were successfully located along with the network dataset.

Distance to Primary Care Clinic

Clinic addresses were geocoded using the methods described above and located to the street network dataset. The OD cost matrix network analysis produced a table of 270913 time and distance variables, representing the length and network distance (miles) between each patient and each clinic. This result was filtered to include only those patient + clinic pairs represented in the LIGHT^2 dataset by cross-referencing a lookup table. Of 9317 patients in the original geocoded dataset, 841 were not included in the lookup dataset, indicating that there was no assigned primary care clinic address included in the file.

Distance to Nearest Grocery Store

Missouri food retailer locations were obtained through the purchase of the ReferenceUSA US Businesses dataset (Infogroup, Inc., 2019).[5] This database was selected as the research literature shows that the ReferenceUSA (formerly InfoUSA) establishment database has a high degree of accuracy compared to other commercial sources. Small retailers were excluded to approximate stores that sell "a wide variety of healthy foods at affordable prices" per previous studies.[6] Grocery retailers were defined as establishments with 10 or more employees having a primary SIC code of 541105 (Grocers-Retail) OR primary NAICS code of 445110 (Supermarkets/Other Grocery, Excluding Convenience Strs). This definition excluded certain establishments that sell a wide variety of fruits and vegetables in addition to general merchandise. Therefore, establishments with having a primary NAICS 531102 (Department Stores) with a listed company name of "Target" or "Walmart" were also included in the grocer establishment dataset. The resulting dataset contained 852 geo-located establishments. An OD cost matrix network analysis was again used to determine travel time and distance to each type of food retailer (ESRI, 2018).[4] The analyses produced matrices of 93167 records (grocery outlets). Results were summarized for each patient to return the minimum values for distance and time.

Physical activity opportunities:

To assess opportunities for physical activity, we generated WalkScore™ values for each patient's census block using the WalkScore™ API (WalkScore, 2019). WalkScore™ is a rating (on a scale of 0 to 100) of the pedestrian friendliness of the area around a specified location.[7] To determine the validity of the WalkScore™ data for our model, we generated a visual map of the WalkScore™ data in Boone county, Missouri. The visual assessment found that addresses in Boone county residential areas with access to safe trails and parks were coded as less/least walkable, indicating that the WalkScore™ dataset may not represent physical activity opportunities in areas with networks of unpaved trails such as our local county. In addition, WalkScore™ data is missing for most rural locations. Hence, we excluded area-level

walkability measures from our models because we could not identify reliable area-level physical activity measures for our suburban and rural patient population cohort.

Social Capital Measure:

Neighborhood (ZIP-code level) social capital was assessed using the number of civic or social organizations per capita, obtained by summarizing data from the 2017 US Census Bureau ZIP code Business Patterns. Civic or social associations are defined by the North American Industry Classification System (NAICS) economic classification system using the definition established by Rupasingha et al. (2006, with updates).[8] The social capital and social isolation measures are components of the validated Social Capital Index by Rupasingha et al. (2006, with updates).[8] Social isolation is measured as percentage of adults living alone at neighborhood-level. However, we included marital status reported in patients' clinical chart as a variable which indicates 43% of our cohort is married, and we have individual-level single or widowed status for our cohort. Hence, we excluded the social isolation measure from our measurement of social capital.

Domestic Violence Injury Rates:

Domestic violence injury rates are counts of emergency room (ER) and hospital inpatient discharges for injuries coded as Spouse/partner abuse. Data were obtained at ZIP code-level from the Missouri Department of Health and Senior Services (DHSS) Missouri Information for Community Assessment (MICA) Injury MICA data profiles query tool. We used multi-year aggregates for years 2011-2015 to avoid data suppression.

**Supplementary File S1-Table 4. Area-level variables**			
**Variable Definition**	**Source(s)**	**Source Time Period**	**Geographic Resolution**
Percentage of Population below 200% Federal Poverty Level	US Census Bureau, American Community Survey 5-Year Estimates (2014)	2010-2014	Census Tract
Educational Attainment – Scaled Variable	US Census Bureau, American Community Survey 5-Year Estimates (2014)	2010-2014	Census Block Group
Travel time to Primary Care > 30 Minutes (Binary)	LIGHT² Patient Dataset<br><br>ESRI, StreetMap North America (2013)	2013-2015<br><br>2010	Address
Distance to Nearest Grocery Store	Infogroup, ReferenceUSA (2019)	2019	Address
Civic and Social Associations Rate (per 100,000)	US Census Bureau, County Business Patterns (2017)	2017	ZIP Code
Domestic violence injury hospitalization rate (per 1,000)	Missouri Department of Health and Senior Services, Injury MICA (2017)	2011-2015	ZIP Code

Supplementary Table S1: Results from Parsimonious model for Diastolic Blood Pressure (DBP) outcome ($R^2 = 0.39$)

**Parameter**	**Adjusted β (95% Confidence Limits)**	**P-value**
Intercept	-8.82 (-19.46, 1.81)	0.10
Pre-intervention BMI	0.078 (0.048, 0.11)	<0.001
Pre-intervention SBP	0.15 (0.08, 0.23)	<0.001
Pre-intervention DBP	0.83 (0.75, 0.91)	<0.001
Female (ref=Male)	-5.33 (-9.75, -0.91)	0.018
Pre-intervention SBP × Female	0.04 (0.01, 0.08)	0.01
Age	0.32 (0.20, 0.45)	<0.001
Pre-intervention SBP × age	-0.003 (-0.004, -0.002)	<0.001
Q1: <5 Nurse care contacts (ref=>21)		
Q2: 5-10 Nurse care contacts	-0.55 (-1.18, 0.09)	0.09
Q3: 11-21 Nurse care contacts	-0.07 (-0.70, 0.54)	0.81
Q4: >22 Nurse care contacts	-0.87 (-1.50, -0.25)	0.006
Smoker Yes (ref=No)	0.86 (0.28, 1.44)	0.003
Smoking status missing (ref=No)	-1.23 (-3.22, 0.75)	0.22
High risk Alcohol use	2.43 (0.76, 4.12)	0.004
Number of comorbidities	0.06 (0.008, 0.11)	0.02
Female Sex × mood_disorder	1.63 (0.55, 2.98)	0.0029
Proportion not graduated HS in the area, Q4 >14.06% (ref)		
Proportion not graduated HS in the area, Q1 <3.17%	-0.69 (-1.34, -0.04)	0.04
Proportion not graduated HS in the area, Q2 3.17-8.79 %	-0.79 (-1.44, -0.14)	0.02
Proportion not graduated HS in the area, Q3 8.79- 14.06%	0.0002 (-0.66, 0.66)	0.99
Percentage of population below 200% of the FPL (mean, SD) for patient's census-tract	0.20 (0.05, 0.35)	0.008
Pre-intervention DBP × Poverty proportion below 200%FPL	-0.003 (-0.0045, -0.0006)	0.008
Domestic violence injury rates (per 1,000) for patient zip codes, 2011-2015	0.96 (0.34, 1.58)	0.002

DBP=diastolic blood pressure, BMI=body mass index (kg/m^2), SBP=systolic blood pressure, ref=reference category, HS = High School FPL=Federal poverty level; × =interaction symbol; R^2=unadjusted R^2, coefficient of determinations

**Supplementary Table S2: Multivariable models of LDL cholesterol outcome for 2377 participants with pre- and post-intervention measures.**									
	**Model 1 (R^2 0.289)**			**Model 2 (R^2 0.296)**			**Model 3 (R^2 0.298)**		
**Parameter**	**β**	**SE**	**p-value**	**β**	**SE**	**p-value**	**β**	**SE**	**p-value**
Intercept	64.33	6.18	<0.001	67.99	6.62	<.0001	68.21	7.05	<0.001
Pre-intervention BMI	-0.24	0.085	0.005	-0.201	0.085	.018	-0.19	0.085	0.02
Pre-intervention LDL	0.56	0.02	<0.001	0.56	0.02	<.001	0.56	0.02	<0.001
Female (ref=Male)	7.87	1.34	<0.001	7.72	1.34	<.001	7.87	1.34	<0.001
Non-White race (ref=White)	-4.16	1.91	0.04	-3.21	1.94	.09	-3.24	1.96	0.09
Age	-0.28	0.06	<0.001	-0.28	0.06	<.0001	-0.28	0.06	<0.001
Marital Status (ref=married)									
Single	-1.74	1.88	0.35	-0.66	1.90	.73	-0.41	1.91	0.83
Widowed	-2.05	1.88	0.28	-1.50	1.88	.42	-1.35	1.88	0.47
Other	0.52	1.98	0.79	1.72	1.99	.39	1.88	1.20	0.35
High healthcare resource utilizer (ref=low utilizer)	-2.07	2.18	0.34	-2.12	2.18	.33	-1.82	2.18	0.40
Mood disorder present (ref=absent or unknown)	-1.56	1.79	0.38	-1.75	1.79	.33	-1.73	1.70	0.34
High risk alcohol use (ref=unknown)	-3.88	6.09	0.52	-3.53	6.07	.56	-3.45	6.07	0.57
Nurse care contacts (ref=Q1: <5)									
Quartile 2: 5-10	1.89	1.86	0.31	1.77	1.86	.34	1.74	1.86	0.39
Quartile 3: 11-22	0.31	1.78	0.86	0.20	1.78	.91	0.05	1.78	0.97
Quartile 4: >22	0.37	1.81	0.83	0.45	1.81	.80	0.27	1.81	0.88
Current smoker (ref=No)									
Yes	-1.85	1.80	0.30	-0.82	1.81	.65	-0.77	1.81	0.67
Unknown	-9.97	6.29	0.11	-9.85	6.27	.12	-9.78	6.29	0.12
Number of comorbidities	-0.48	0.18	0.007	-0.42	0.18	.017	-0.40	0.18	0.026
Proportion area residents who did not graduate from high school (ref=Q4: >14.06%)									
Quartile 1: <3.17%				1.40	1.90	.46	1.54	2.02	0.44
Quartile 2: 3.17-8.79%				2.57	1.91	.18	2.48	1.93	0.19
Quartile 3: 8.79-14.06%				-0.18	1.95	.93	-0.28	1.97	0.88
Percentage of area population below 200% of the FPL				-0.12	0.05	.01	-0.11	0.05	0.031
Domestic violence injury rates (per 1,000) for patient zip codes				-5.22	1.82	.004	-5.41	1.87	0.004
Civic and Social Associations Rate (per 100,000 population)				-0.07	0.13	0.58	-0.07	0.13	0.58
Distance to nearest grocery store							0.14	0.19	0.47

Driving time to PCP (ref=<=30)									
>30 minutes							-0.11	1.80	0.95
Unknown							-4.05	1.94	0.04

β = regression coefficients; SE = Standard Error; BMI = Body Mass Index; LDL=low density lipoprotein; ref=reference category, FPL=Federal poverty level; PCP=primary care provider;× =interaction symbol; R^2=coefficient of determinations

**Supplementary Table S3: Multivariable models of hemoglobin A1C outcome for 1290 participants with pre-and post-intervention measures.**									
	**Model 1 (R^2 0.396)**			**Model 2 (R^2 0.399)**			**Model 3 (R^2 0.40)**		
**Parameter**	**β**	**SE**	**p-value**	**β**	**SE**	**p-value**	**β**	**SE**	**p-value**
Intercept	4.08	0.43	<.001	4.11	0.45	<.001	3.88	0.46	<0.001
Pre-intervention BMI	0.001	0.004	0.75	0.0005	0.004	0.89	0.0004	0.004	0.92
Pre-intervention A1C	0.52	0.04	<.001	0.51	0.04	<.001	0.51	0.04	<0.001
Female (ref=male)	-1.24	0.34	<.001	-1.25	0.34	<.001	-1.28	0.34	<0.001
Pre-intervention A1C × female sex	0.18	0.05	<.001	0.18	0.05	<.001	0.19	0.048	<0.001
Non-White race (ref=White)	-1.17	0.40	0.004	-1.16	0.40	0.004	-1.159	0.40	<0.004
Pre-intervention A1C × non-White race	0.14	0.05	0.014	0.14	0.05	0.014	0.14	0.06	0.011
Age	-0.006	0.003	0.05	-0.006	0.003	0.06	-0.007	0.003	0.05
Marital status (ref=married)									
Single	-0.04	0.097	0.70	-0.02	0.10	0.85	-0.009	0.10	0.93
Widowed	-0.04	0.09	0.67	-0.04	0.09	0.66	-0.04	0.09	0.66
Other	-0.10	0.10	0.34	-0.09	0.10	0.37	-0.09	0.105	0.41
High healthcare resource utilizer (ref=low utilizer)	-0.024	0.10	0.82	-0.02	0.10	0.82	-0.002	0.10	0.98
Mood disorder present (ref=absent or unknown)	-0.06	0.09	0.50	-0.05	0.09	0.59	-0.05	0.09	0.55
High risk alcohol use (ref=unknown)	-0.24	0.28	0.40	-0.25	0.28	0.37	-0.27	0.28	0.35
Nurse care contacts (ref=Q1: <5)									
Quartile 2: 5-10	-0.18	0.098	0.06	-0.19	0.098	0.051	-0.19	0.098	0.06
Quartile 3: 11-21	-0.14	0.09	0.12	-0.14	0.09	0.13	-0.14	0.09	0.13
Quartile 4: >21	-0.13	0.09	0.16	-0.13	0.094	0.17	-0.11	0.09	0.22
Current smoker (ref=No)									
Yes	-0.18	0.09	0.05	-0.19	0.09	0.04	-0.19	0.09	0.04
Unknown	-0.54	0.29	0.06	-0.53	0.296	0.07	-0.56	0.296	0.058
Number of comorbidities	0.003	0.008	0.71	0.002	0.009	0.78	0.002	0.009	0.79
Proportion area residents who did not graduate from high school (ref=Q4: >14.06%)									
Quartile 1: <3.17%				-0.022	0.099	0.83	0.02	0.10	0.85
Quartile 2: 3.17-8.79%				0.12	0.10	0.23	0.14	0.10	0.15
Quartile 3: 8.79-14.06%				0.18	0.10	0.07	0.21	0.10	0.035
Percentage of area population below 200% of the FPL				-0.002	0.002	0.47	-0.002	0.003	0.44
Interpersonal violence rates				0.05	0.09	0.56	0.10	0.10	0.29
Civic and Social Associations Rate (per 100,000 population)				0.02	0.007	0.02	0.02	0.007	0.02
Distance to nearest grocery store							0.008	0.01	0.41
Driving time to PCP (ref=<=30)									
Unknown							0.09	0.09	0.34
>30 minutes							0.019	0.09	0.83

β = Regression coefficient; SE = Standard Error; BMI=body mass index (kg/m^2), A1C=hemoglobin A1C, ref=reference category; FPL=Federal poverty level; PCP=primary care provider; × =interaction symbol; R^2=unadjusted R^2, coefficient of determinations

Supplementary Table S4: Multivariable models of systolic blood pressure outcome for 4618 participants with pre-and post-intervention measures									
	**Model 1 (R^2 0.43)**			**Model 2 (R^2 0.43)**			**Model 3 (R^2 0.43)**		
**Parameter**	**β**	**SE**	**p-value**	**β**	**SE**	**p-value**	**β**	**SE**	**p-value**
Intercept	13.47	7.82	0.08	-2.096	9.62	0.83	-3.17	9.66	0.74
Pre-intervention BMI	0.11	0.026	<.001	0.097	0.026	<0.001	0.09	0.026	<0.001
Pre-intervention SBP	0.91	0.07	<.001	0.93	0.07	<0.001	0.93	0.07	<0.001
Pre-intervention DBP	-0.12	0.034	0.001	0.05	0.07	0.48	0.05	0.07	0.49
Female (ref=male)	-8.16	3.95	0.04	-9.06	3.95	0.02	-8.93	3.95	0.02
Pre-intervention SBP × Female sex	0.061	0.03	0.04	0.067	0.029	0.02	0.067	0.03	0.02
Non-White race (ref=White)	0.091	0.59	0.88	0.15	0.60	0.79	0.31	0.60	0.60
Age	0.31	0.11	0.006	0.35	0.11	0.001	0.35	0.11	0.002
Pre-intervention SBP × age	-0.002	0.0009	0.02	-0.002	0.0009	0.008	-0.002	0.0009	0.009
Marital Status (ref=married)									
Single	-0.48	0.57	0.40	-0.51	0.58	0.38	-0.42	0.58	0.47
Widowed	0.91	0.58	0.12	0.78	0.58	0.18	0.81	0.58	0.16
Other	0.72	0.62	0.24	0.50	0.62	0.42	0.54	0.62	0.39
High healthcare resource utilizer (ref=low utilizer)	-0.19	0.57	0.74	-0.14	0.57	0.80	-0.11	0.57	0.85
Mood disorder present (ref=absent or unknown)	-0.26	0.51	0.60	-0.29	0.51	0.57	-0.26	0.51	0.61
High risk alcohol use (ref=unknown)	1.72	1.50	0.25	1.68	1.50	0.26	1.68	1.49	0.26
Nurse care contacts (ref=Q1 <5)									
Quartile 2: 5-10	-0.88	0.57	0.12	-0.80	0.57	0.16	-0.80	0.57	0.16
Quartile 3: 11-22	-0.60	0.55	0.28	-0.58	0.55	0.29	-0.57	0.55	0.30
Quartile 4: >22	-1.18	0.56	0.03	-1.19	0.56	0.03	-1.17	0.56	0.04
Current smoker (ref=No)									
Yes	0.79	0.51	0.12	0.41	0.52	0.43	0.38	0.52	0.47
Unknown	-1.30	1.77	0.46	-1.44	1.77	0.41	-1.49	1.77	0.40
Number of comorbidities	0.11	0.05	0.02	0.10	0.05	0.05	0.099	0.05	0.05
Proportion area residents who did not graduate from high school (ref=Q4: >14.06%)									
Quartile 1: <3.17%				-1.34	0.58	0.02	-1.006	0.61	0.09
Quartile 2:3.17-8.79 %				-0.72	0.58	0.21	-0.57	0.58	0.33
Quartile 3: 8.79-14.06%				0.27	0.59	0.64	0.41	0.59	0.48
Proportion of population below 200% of FPL				0.36	0.13	0.006	0.36	0.13	0.007
Pre-intervention DBP × percentage of population below 200% of the FPL				-0.005	0.002	0.007	-0.005	0.002	0.008
Domestic violence injury rates (per 1,000) for patient zip codes				1.77	0.55	0.001	2.045	0.57	<0.001
Civic and Social Associations Rate (per 100,000 population)				0.06	0.04	0.17	0.06	0.04	0.17
Distance to nearest grocery store							0.043	0.06	0.47

Driving time to PCP (ref=<=30)									
>30 minutes							0.57	0.55	0.29
Unknown							0.53	0.54	0.33

BMI=body mass index (kg/m^2), SBP=systolic blood pressure, DBP=diastolic blood pressure, PCP=primary care provider, ref=reference category, FPL=Federal poverty level; × =interaction symbol; R^2= unadjusted R^2, coefficient of determinations

Supplementary Table S5: Multivariable models of diastolic blood pressure outcome for 4619 participants with pre-and post-intervention measures.

	Model 1 (R^2 0.39)			Model 2 (R^2 0.39)			Model 3 (R^2 0.39)		
**Parameter**	**β**	**SE**	**p-value**	**β**	**SE**	**p-value**	**β**	**SE**	**p-value**
Intercept	0.58	4.48	0.89	-7.47	5.51	0.17	-7.81	5.54	0.16
Pre-intervention BMI	0.08	0.015	<0.001	0.07	0.015	<0.001	0.078	0.015	<0.001
Pre-intervention SBP	0.14	0.038	<0.001	0.15	0.038	0.001	0.15	0.039	<0.001
Pre-intervention DBP	0.73	0.019	<0.001	0.82	0.041	<0.001	0.82	0.041	<0.001
Female (ref=Male)	-4.89	2.26	0.03	-5.43	2.26	0.016	-5.42	2.26	0.016
Pre-intervention SBP × Female	0.041	0.017	0.017	0.045	0.017	0.008	0.045	0.02	0.009
Non-White race (ref=White)	-0.61	0.34	0.06	-0.58	0.34	0.09	-0.55	0.34	0.113
Age	0.28	0.06	<0.001	0.31	0.06	<0.001	0.31	0.06	<.001
Pre-intervention SBP × age	-0.002	0.0005	<0.001	-0.002	0.0005	<0.001	-0.002	0.0005	<.001
High Healthcare Resource Utilizer (ref Low Utilizer)	-0.10	0.33	0.76	-0.054	0.33	0.89	-0.063	0.33	0.85
Nurse care contacts (ref=Q1: <5)									
Quartile 2: 5-10	-0.60	0.33	0.07	-0.55	0.32	0.09	-0.56	0.32	0.08
Quartile 3: 11-21	-0.09	0.31	0.78	-0.09	0.31	0.77	-0.10	0.31	0.74
Quartile 4: >21	-0.89	0.32	0.005	-0.88	0.32	0.005	-0.88	0.32	0.006
Smoker Yes (ref=No)	1.01	0.29	<0.001	0.82	0.30	0.006	0.80	0.30	0.007
Smoking status missing	-1.20	1.02	0.24	-1.25	1.01	0.22	-1.23	1.01	0.22
Marital Status (ref=married)									
Single	0.016	0.33	0.96	-0.005	0.33	0.99	0.018	0.33	0.95
Widowed	-0.35	0.33	0.28	-0.40	0.33	0.22	-0.39	0.33	0.23
Other	0.76	0.35	0.031	0.66	0.35	0.06	0.68	0.36	0.06
Mood Disorder present (ref= absent or unknown)	-0.37	0.29	0.19	-0.39	0.29	0.18	-0.39	0.29	0.18
High risk Alcohol use (ref Alcohol use unknown)	2.41	0.86	0.005	2.39	0.85	0.005	2.44	0.86	0.004
Number of comorbidities	0.08	0.03	0.005	0.07	0.03	0.010	0.07	0.03	0.011
Proportion not graduated high school in the area, Q4 >14.06% (ref)									
Q1 <3.17%				-0.68	0.33	0.041	-0.59	0.35	0.08
Q2 3.17-8.79 %				-0.79	0.33	0.017	-0.74	0.33	0.028
Q3 8.79- 14.06%				0.001	0.33	0.99	0.03	0.34	0.93

Percentage of area population below 200% of the FPL				0.18	0.076	0.014	0.19	0.07	0.014
Pre-intervention DBP*Proportion below 200FPL				-0.002	0.001	0.015	-0.0024	0.001	0.016
Domestic violence injury rates (per 1,000) for zip codes				0.94	0.32	0.003	1.011	0.33	0.002
Civic and Social Associations Rate (per 100,000 population)				0.012	0.02	0.61	0.012	0.02	0.61
Distance to nearest grocery store							0.05	0.034	0.14
Time to PCP<=30 (ref)									
Time to PCP>30							-0.26	0.31	0.40
Time to PCP unknown							0.16	0.31	0.60

β = Regression coefficient; SE = Standard Error; BMI=body mass index (kg/m^2), SBP=systolic blood pressure, DBP=diastolic blood pressure, ref=reference category, FPL=Federal poverty level; PCP = primary care provider, × =interaction symbol; R^2=unadjusted R^2, coefficient of determinations

Supplementary Table S6: Solution for Random Effects by clinics- LDL outcome

Effect	Clinic Name	Estimate	Standard Error of Prediction	DF	t Value	Pr > |t|
**clinic**	**Fairview**	-0.17	1.20	2035	-0.14	0.88
**clinic**	**Fayette**	0.088	1.45	2035	0.06	0.95
**clinic**	**Fulton**	-1.89	1.25	2035	-1.5	0.13
**clinic**	**Green Meadows**	0.14	1.18	2035	0.12	0.90
**clinic**	**Keene**	0.46	1.43	2035	0.32	0.75
**clinic**	**Smiley**	0.063	1.37	2035	0.05	0.96
**clinic**	**Woodrail**	1.31	1.17	2035	1.12	0.26

Covariance Parameter Estimates

Covariate Parameter	Estimate
**clinic**	2.48
**Residual**	916.93

Supplementary Table S7: Solution for Random Effects by clinics: HbA1C outcome

Effect	Clinic Name	Estimate	Standard Error of Prediction	DF	t Value	Pr > |t|
**clinic**	**Fairview**	-0.09	0.078	1035	-1.18	0.24
**clinic**	**Fayette**	-0.03	0.11	1035	-0.26	0.79
**clinic**	**Fulton**	-0.04	0.09	1035	-0.51	0.61
**clinic**	**Green Meadows**	0.17	0.07	1035	2.3	0.02
**clinic**	**Keene**	-0.02	0.10	1035	-0.2	0.84
**clinic**	**Smiley**	-0.04	0.09	1035	-0.5	0.62

**clinic**	**Woodrail**	0.06	0.08	1035	0.73	0.46
**Covariance Parameter Estimates**						
**Cov Parm**			**Estimate**			
**clinic**			0.01469			
**Residual**			1.1994			

**Supplementary Table S8: Solution for Random Effects by clinics: Systolic BP outcome**						
**Effect**	**Clinic Name**	**Estimate**	**Standard Error of Prediction**	**DF**	**t Value**	**Pr > |t|**
**clinic**	**Fairview**	-2.45	0.73	3805	-3.36	<0.001
**clinic**	**Fayette**	-0.27	0.96	3805	-0.29	0.77
**clinic**	**Fulton**	0.50	0.78	3805	0.64	0.52
**clinic**	**Green Meadows**	-0.07	0.68	3805	-0.1	0.92
**clinic**	**Keene**	-0.02	0.91	3805	-0.02	0.98
**clinic**	**Smiley**	1.46	0.82	3805	1.77	0.08
**clinic**	**Woodrail**	0.86	0.68	3805	1.26	0.20
**Covariance Parameter Estimates**						
**Covariate parameter**			**Estimate**			
**clinic**			1.9578			
**Residual**			171.92			

**Supplementary Table S9: Solution for Random Effects by clinics: Diastolic BP outcome**						
**Effect**	**Clinic Name**	**Estimate**	**Standard Error of Prediction**	**DF**	**t Value**	**Pr > |t|**
**clinic**	**Fairview**	-1.91	0.46	3806	-4.13	<0.001
**clinic**	**Fayette**	0.55	0.6038	3806	0.92	0.36
**clinic**	**Fulton**	0.15	0.4926	3806	0.3	0.77
**clinic**	**Green Meadows**	0.33	0.4355	3806	0.76	0.45
**clinic**	**Keene**	0.12	0.57	3806	0.21	0.83
**clinic**	**Smiley**	0.44	0.5162	3806	0.85	0.39
**clinic**	**Woodrail**	0.33	0.4382	3806	0.74	0.46
**Covariance Parameter Estimates**						
**Covariate parameter**			**Estimate**			
**clinic**			0.883			
**Residual**			57.357			

Supplementary Figure S1: Interaction of female sex with pre-intervention systolic blood pressure (BP) on adjusted change in systolic BP

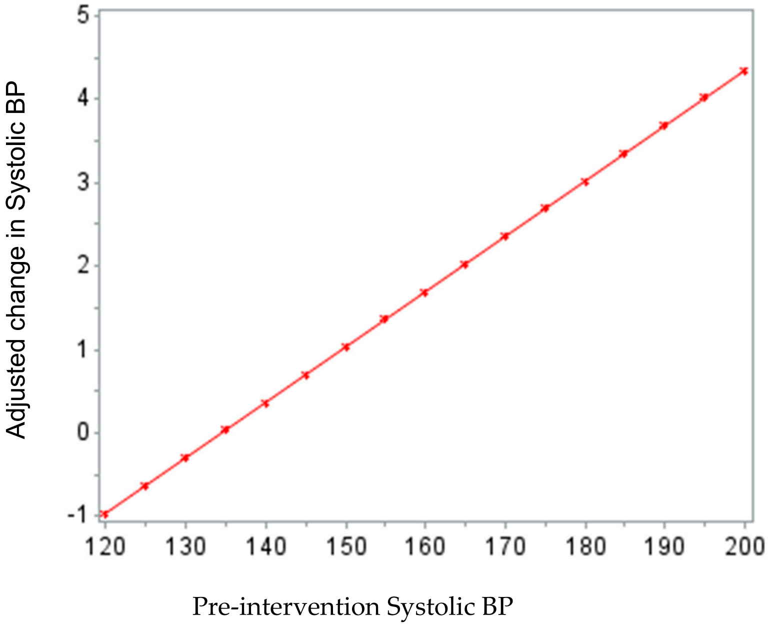

Supplementary Figure S2: Interaction of female sex with pre-intervention systolic blood pressure (BP) on adjusted change in diastolic BP

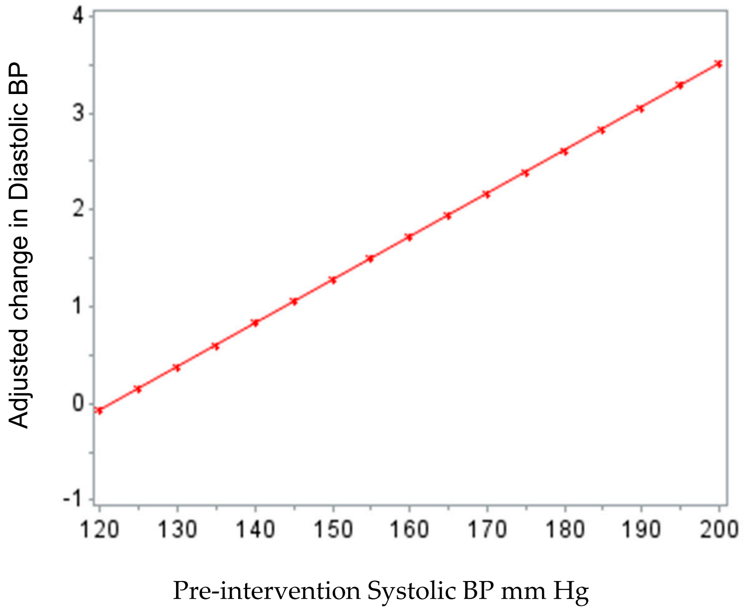

Supplementary Figure S3: Interaction of female sex with pre-intervention HbA1C on adjusted change in HbA1C

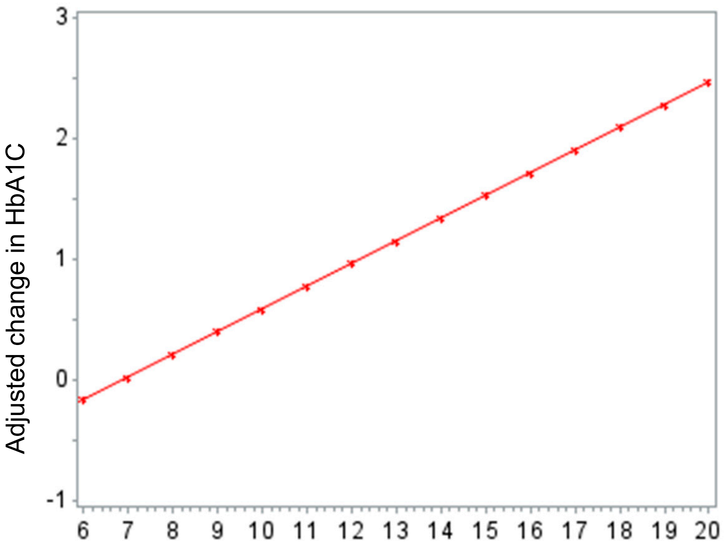

References:

1. ESRI: ArcGIS Online World Geocoding Service. In. Redlands, CA: Environmental Systems Research Institute; 2019.
2. 2014 TIGER/Line Shapefiles (machine-readable data files) [https://www.census.gov/geographies/mapping-files/time-series/geo/tiger-line-file.2014.html]
3. 2010-2014 American Community Survey 5-year Estimates [Data File] [https://www2.census.gov/programs-surveys/acs/summary_file/2014/data/5_year_entire_sf/]
4. ESRI: ArcGIS Desktop: Release 10. In. Redlands, CA: Environmental Systems Research Institute; 2018.
5. ReferenceUSA [dataset] [http://www.referenceusa.com]
6. Low-income and low-supermarket-access census tracts, 2010-2015 [https://www.ers.usda.gov/publications/pub-details/?pubid=82100]
7. Walk Score API Sign Up [https://www.walkscore.com/professional/api-sign-up.php]
8. Rupasingha A, Goetz SJ, Freshwater D: The production of social capital in US counties. *The Journal of Socio-Economics* 2006, 35(1):83-101.
